# Lead‐Free Tin‐Based Perovskite LEDs Toward Rec. 2020: Organic Anion Coordination for Oxidation Suppression

**DOI:** 10.1002/advs.202511006

**Published:** 2025-09-16

**Authors:** Seungjae Lee, Heeseung Lee, Joonho Park, Hyeonwoo Yeo, Junho Kim, Changjo Kim, Hyojun Kim, Seyun Lee, Jihyung Lee, Yun Hoo Kim, Seungbok Lee, Seonju Jeong, Wu Bin Ying, Ryong‐Gyu Lee, Yong‐Hoon Kim, Jung‐Yong Lee

**Affiliations:** ^1^ School of Electrical Engineering (EE) Korea Advanced Institute of Science and Technology (KAIST) Yuseong‐gu, 291 Daehak‐ro Daejeon 34141 Republic of Korea; ^2^ Graduate School of Semiconductor Technology (ST) Korea Advanced Institute of Science and Technology (KAIST) Yuseong‐gu, 291 Daehak‐ro Daejeon 34141 Republic of Korea

**Keywords:** alkali metals, defect passivation, high color purity, lead‐free perovskites, organic anions, self‐oxidation, tin metal‐free

## Abstract

Lead halide perovskites are highly promising for optoelectronic applications, but the toxicity of lead (Pb) ions presents significant health and environmental challenges. Recent efforts to replace Pb metal with tin (Sn) face challenges due to Sn's oxidation instability, limiting its use in perovskite light‐emitting diodes (PeLEDs). While Sn metal additives are traditionally utilized to mitigate the oxidation of Sn^2+^, alternative stabilization strategies remain unexplored. In this study, an organic anion‐coordination for oxidation suppression (OCOS) strategy is introduced, which effectively stabilizes Sn‐based perovskites. By incorporating alkali metal–organic anions, OCOS significantly enhances external quantum efficiency (EQE) and luminance. The organic anions coordinate with Sn^2+^ via lone pair electron interactions, while alkali metals inhibit Sn vacancy formation, further enhancing film quality and device performance. Moreover, the energetic stabilization induced by the OCOS strategy is quantified by density functional theory (DFT) calculations and clarify its mechanism in terms of electronic structural change. Using this strategy, an EQE of 10.01% is achieved at an emission wavelength of 638 nm in lead‐free Sn‐based PeLEDs. This work provides new insights into Sn stabilization strategies and advances the development of lead‐free perovskite optoelectronic devices.

## Graphical abstract

This study introduces an organic anion‐coordination for oxidation suppression (OCOS) strategy using alkali metal–organic anions (e.g., KEtO) to stabilize Sn^2+^ in lead‐free Sn‐based perovskites for PeLEDs. By coordinating with Sn^2+^ and inhibiting vacancies, OCOS enhances film quality, EQE (to 10.01% at 638 nm), and luminance (to 139.9 cd m^−2^), outperforming traditional Sn metal additives, as validated by DFT and experiments.

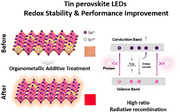

## 1 Introduction

Solution‐processed metal‐halide perovskites have attracted significant attention in information displays and energy applications owing to their exceptional optoelectronic properties, flexibility, and cost‐effectiveness.^[^ ^1^, ^2^, ^3^, ^4^, ^5^ ^]^ Notably, recent advancements have enabled efficient perovskite‐based light‐emitting diodes (PeLEDs) to achieve external quantum efficiency (EQE) exceeding 20%.^[^ ^6^, ^7^ ^]^ However, the prevalent use of lead (Pb) in these perovskites raises critical concerns regarding Pb toxicity and regulatory restrictions, such as the Restriction of Hazardous Substances Directive (RoHS), which hinder their commercial adoption. There has been a growing focus on identifying eco‐friendly alternatives to Pb, with particular emphasis on elements such as germanium (Ge), tin (Sn), copper (Cu), bismuth (Bi), antimony (Sb), and other potential divalent cation replacements.^[^ ^8^, ^9^ ^]^

Substituting lead, however, requires careful balancing between material stability and device performance. For instance, Bi‐based double perovskites exhibit remarkable stability in ambient environments, but their indirect bandgap of ≈2 eV significantly limits their optoelectronic applications.^[^ ^10^, ^11^ ^]^ Additionally, both Bi and Sb, typically in trivalent states (i.e., Bi^3+^ and Sb^3+^), induce layered vacant structures that hinder efficient charge transport.^[^ ^11^ ^]^ Ge, with its 4s^2^ configuration, tends to lose non‐bonding electron pairs readily, leading to chemical instability. Moreover, its poor solubility in common polar organic solvents further poses significant challenges for solution processing.^[^ ^12^ ^]^

Among lead‐free perovskite alternatives, Sn is particularly favored for its exceptional optoelectrical properties, despite being less stable than Bi and Sb.^[^ ^13^, ^14^ ^]^ However, the performance of Sn‐based perovskite LEDs remains inferior to their Pb‐based counterparts, primarily due to the high defect density caused by rapid crystallization, which detrimentally influences luminescence.^[^ ^15^ ^]^ To overcome these challenges, various strategies have been explored, including additive engineering,^[^ ^16^, ^17^ ^]^ low‐dimensional structures,^[^ ^18^, ^19^ ^]^ and solvent modifications^[^ ^20^ ^]^ to delay crystallization and improve film quality.

A critical problem that needs to be resolved is the spontaneous oxidation of Sn^2+^ to Sn^4+^. Conventionally, the addition of Sn metal powder into the precursor solution has been employed to suppress Sn oxidation,^[^ ^21^, ^22^ ^]^ thereby improving both the performance and stability of Sn‐based PeLEDs.^[^ ^21^ ^]^ However, this approach often leads to excessive tin precipitation within the precursor, reducing its effectiveness in terms of device performance.^[^ ^23^ ^]^

Here, we introduce an organic anion‐coordination for oxidation suppression (OCOS) strategy employing alkali metal–organic anion complexes to effectively suppress Sn^2+^ oxidation in Sn‐based perovskites. Uncoordinated Sn^2+^ ions readily oxidize to stable Sn^4+^, but the electron‐donating ethoxide anion (EtO or C_2_H_5_O) facilitates the formation of C_2_H_5_O― Sn complexes, thereby suppressing this redox instability. Unlike carboxylate or sulfonate functional groups, which have been frequently employed in previous studies but possess relatively weak electron‐donating properties due to their strongly acidic conjugate acids, the ethoxide anion derived from ethanol acts as a much stronger electron‐donating property. This allows it to coordinate more effectively with Sn^2+^, providing enhanced protection against oxidation. Simultaneously, this approach passivates X‐site halide vacancies, which could otherwise act as non‐radiative recombination centers, thereby enhancing luminance and external quantum efficiency (EQE). Moreover, prior researches indicate that alkali metals raise the formation energy of Sn vacancies, improve tolerance to defects, and simultaneously passivate A‐site vacancies.^[^ ^23^, ^24^ ^]^ Additionally, they form I_3_^−^―M^+^(metal ion) complexes in the Sn‐based perovskite precursor, limiting Sn‐induced iodine trimer defects and trap‐assisted recombination.^[^ ^24^, ^25^ ^]^

Perovskite LEDs employing the OCOS strategy demonstrated marked improvements in EQE and luminance compared to devices utilizing Sn metal.^[^ ^16^, ^22^, ^23^ ^]^ Specifically, luminance increased from 11.85 to 139.9 cd m^−2^ and EQE from 0.54% to 10.01%. These results highlight the potential of the OCOS strategy as an effective alternative for the development of high‐performance lead‐free perovskite LEDs.

## 2 Results and Discussion

### 2.1 Organic Anion‐Coordination‐Induced Oxidation Suppression (OCOS)

**Figure 1:**
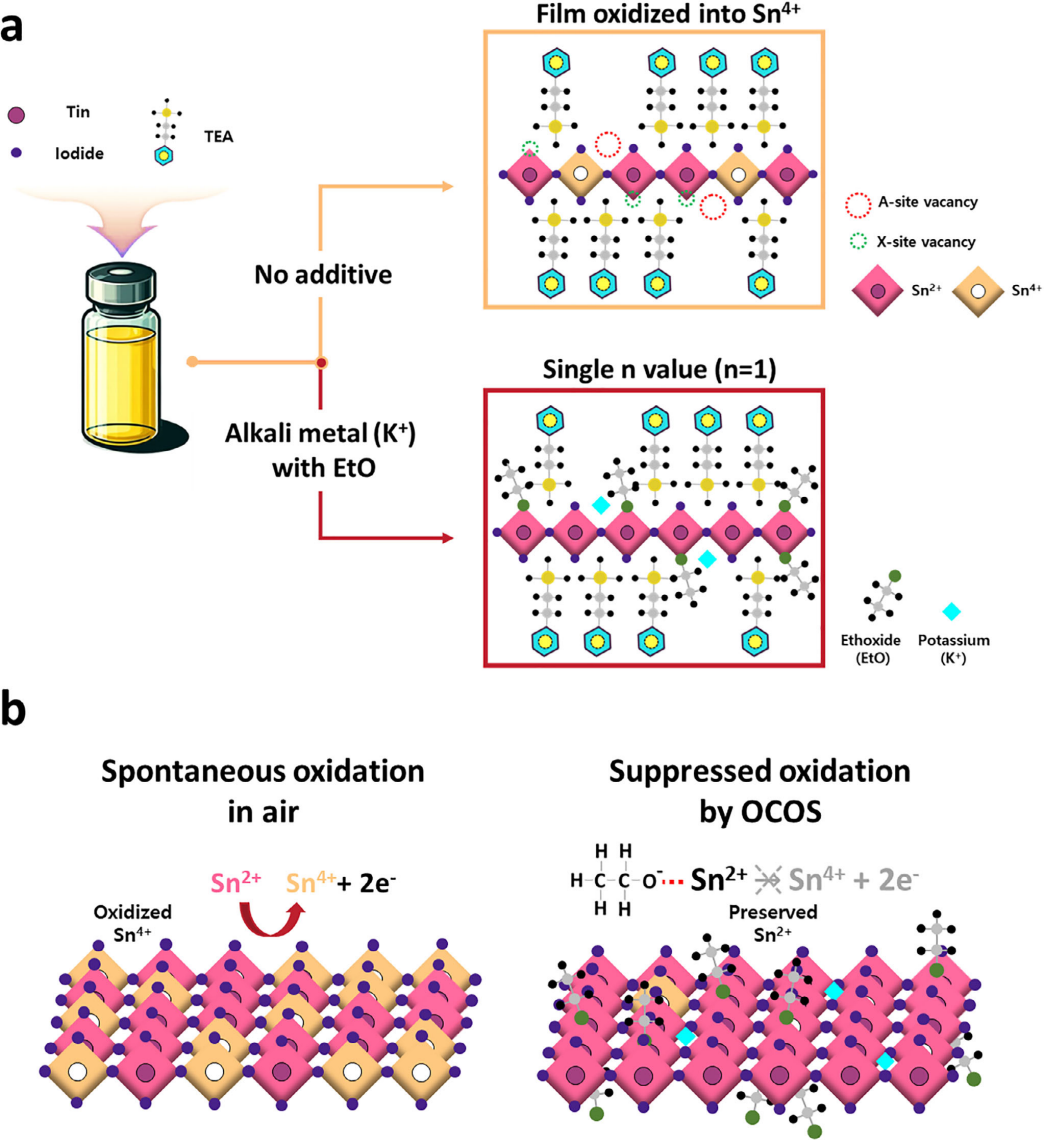
a) Schematic representation of variations in perovskite crystal structures induced by alkali metal (K^+^) ― organic anion (ethoxide, EtO^−^) complex in contrast to the control perovskite film. b) Schematic illustrating the oxidation suppression mechanism of the organic anion (EtO^−^).

We prepared lead‐free 2D perovskite films by combining thiophenethylammonium iodide (TEAI) and tin iodide (SnI_2_) at a stoichiometric 2:1 molar ratio, maintaining a Sn^2+^ concentration of 0.3 M in a dimethylformamide (DMF)/dimethyl sulfoxide (DMSO) solvent mixture to form (TEA)_2_SnI_4_ perovskite.^[^ ^14^, ^26^ ^]^ As shown in **Figure** **1**a, pristine additive‐free perovskite films exhibited a 2D perovskite structure with a single octahedral layer (*n* = 1) and a noticeably higher proportion of Sn^4+^ arising from Sn^2+^ oxidation. To mitigate this redox instability, we implemented the OCOS strategy by incorporating ethoxide (EtO^−^) organic anions with alkali metal potassium (K^+^), referred to as KEtO. Potassium ion (K^+^) was selected over other alkali metal candidates (Li^+^, Na^+^, Cs^+^) for its exceptional color purity, evidenced by a narrower full width at half maximum (FWHM) and a distinct electroluminescence (EL) peak, and its superior device performance, which will be addressed in later sections. EtO^−^ effectively donates lone pair electrons to Sn^2+^, suppressing its oxidation (Figure 1b) compared to pristine films without additives as depicted in Figure 1a.

For optimal optical properties and stability in perovskite LEDs, it is crucial to retain a low dimensional 2D structure.^[^ ^27^, ^28^, ^29^ ^]^ As illustrated in Figure S1 (Supporting Information), replacing potassium (K^+^) with cesium (Cs^+^) results in a mixed‐phase composition comprising both *n* = 1 and *n* ≥ 2 phases, indicating that Cs^+^ may be unsuitable for PeLEDs. Phase variation arises from disparities in the ionic radii of alkali metals. Specifically, K^+^ ions, which can partially replace TEA^+^, are too small to induce additional phases, thereby preserving the low‐dimensional structure confined to the edges of the 2D perovskite lattice or grain boundaries. In contrast, Cs^+^ ions are large enough to integrate into the perovskite lattice and actively participate in A‐site coordination, leading to a structural transformation (i.e., *n* ≥ 2). Furthermore, the incorporation of Cs⁺ between layers increases the effective A‐site ionic radius, potentially raising the Goldschmidt tolerance factor (*t*) beyond the stable range (0.8–1.0),^[^ ^27^ ^]^ destabilizing the structure and promoting structural reconfiguration. Figure S2 (Supporting Information) compares the absorption spectra, revealing that PeKEtO maintained a single‐layered (*n* = 1) structure similar to the control, whereas PeCsEtO displayed an additional peak at 680 nm, confirming the formation of multilayered (*n* ≥ 2) octahedral phases.^[^ ^30^, ^31^ ^]^ X‐ray diffraction (XRD) analysis in Figure S3 (Supporting Information) shows that the control and PeKEtO films exhibit multiple peaks characteristic of conventional 2D perovskites, while PeCsEtO displays a broadened (002) peak, with its FWHM increasing from 0.29° to 0.38°, attributed to increased *d*‐spacing from higher inorganic layer density, consistent with Bragg's law.^[^ ^27^, ^30^ ^]^

### 2.2 Analysis of Chemical Property Changes of Perovskite Films by OCOS

**Figure 2:**
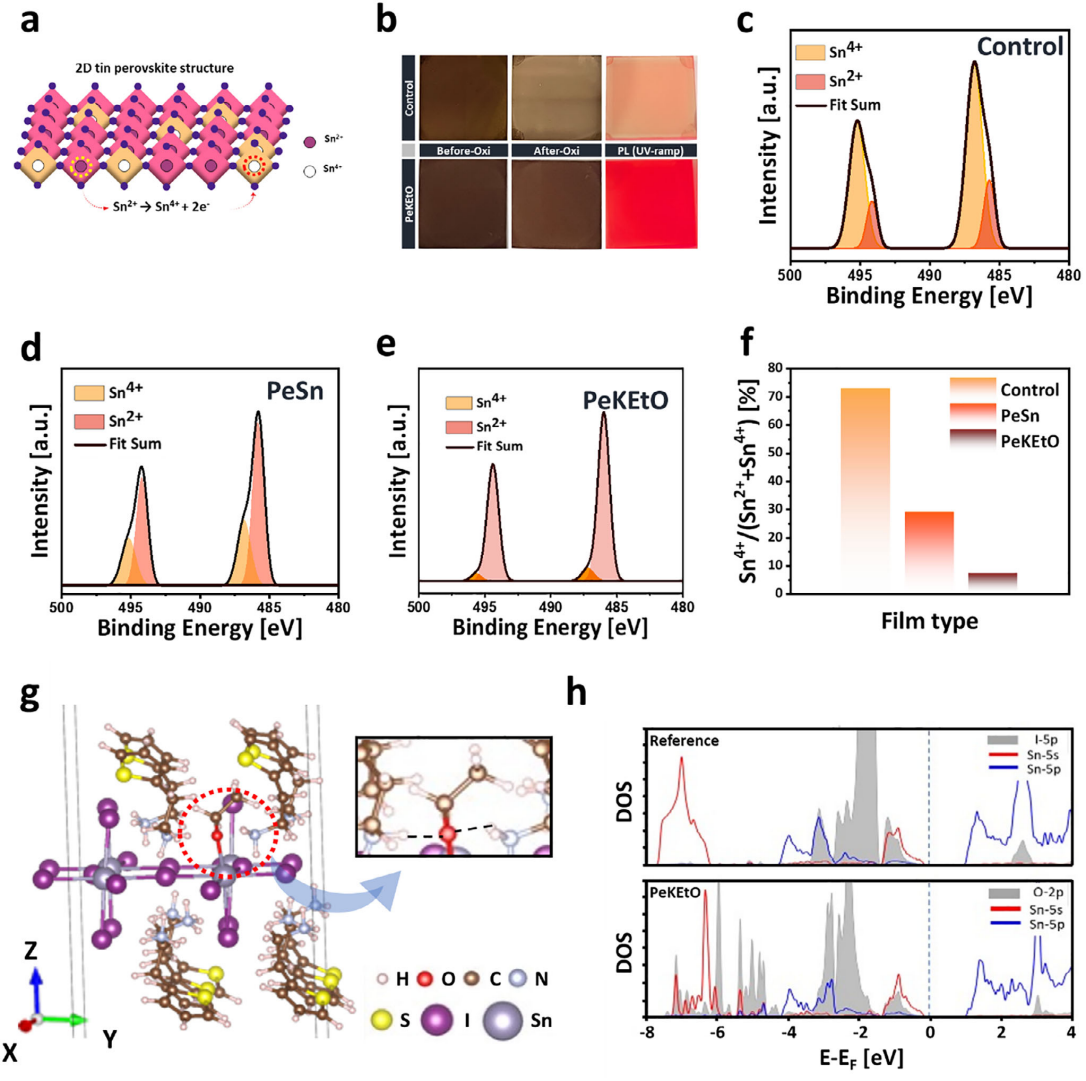
a) Schematic depiction of Sn^2+^ oxidation to Sn^4+^ within a (TEA)_2_SnI_4_ film. b) Table illustrating thin film conditions of control and KEtO‐treated films before and after oxidation, including PL emission under 365 nm UV light. X‐ray photoelectron spectroscopy (XPS) of Sn 3d for c) the control film, d) PeSn, and e) the perovskite film treated with KEtO additive. f) Proportion of Sn^4+^ across various additive treatments. g) DFT‐optimized single‐layer 2D (TEA)_2_SnI_4_ crystal model in which an I^−^ has been substituted by EtO^−^. The black dashed lines in the inset indicate hydrogen bonds between O in EtO^−^ and the H atoms in nearby two TEA^+^. h) PDOS of the pristine (TEA)_2_SnI_4_ and EtO^−^‐passivated (TEA)_2_SnI_4_. Dotted lines denote the valence band maximum reference energy levels.

Tin‐based perovskite materials are highly prone to oxidation, as shown in **Figure** **2**a, because Sn^2+^ is easily oxidized to Sn^4+^ through the loss of electrons from the 5s orbital of Sn.^[^ ^32^ ^]^ This oxidization process disrupts the perovskite lattice by creating Sn vacancies, which act as non‐radiative recombination centers that can significantly diminish photoluminescence (PL).^[^ ^14^, ^32^ ^]^ Compared to lead‐based perovskites, Sn‐based perovskites exhibit considerably lower oxidation stability, making them more susceptible to performance degradation.^[^ ^33^ ^]^ Therefore, suppressing Sn^2+^ oxidation is essential for improving both the optoelectronic performance and long‐term stability of Sn‐based perovskite LEDs.^[^ ^14^, ^32^, ^33^ ^]^

To assess the effectiveness of the OCOS strategy in suppressing Sn^2+^ oxidation, we first compared the photoluminescence (PL) intensity and oxidation tolerance of control films with those treated using either Sn metal addition or OCOS. As shown in Figure 2b, the control films oxidized readily, leading to a marked reduction in PL intensity. In contrast, OCOS‐treated films maintained significantly higher PL intensity, demonstrating enhanced oxidation resistance and enhanced optoelectronic stability.

X‐ray photoelectron spectroscopy (XPS) was employed to verify the successful coordination of ethoxide with the perovskite lattice and to further evaluate the effectiveness of the OCOS strategy in suppressing Sn^2+^ oxidation. The formation of EtO‐Sn complexes was confirmed by Sn 3d XPS analysis (Figure 2c,e), where the Sn^2+^ peak position in the PeKEtO film (494.4 eV) exhibited a 0.3 eV shift toward higher binding energy compared to the control (494.1 eV). This shift is derived from Lewis acid‐base interactions between ethoxide and Sn^2+^, which redistribute the electron density of Sn^2+^ due to the electronegativity differences, resulting in increased effective nuclear charge and consequently higher binding energy without direct electron transfer. Furthermore, analysis of the Sn 3d spectra revealed a significant reduction in Sn^4+^ peaks at ≈487 and 496 eV for both perovskite film treated with Sn metal (PeSn) and perovskite film incorporated with KEtO (PeKEtO) compared to the control film without additives (Figure 2c–e). Notably, the PeKEtO film exhibited even lower Sn^4+^ content than the PeSn film (Figure 2d,e). Quantitatively, the Sn^4+^ ratio to total Sn ions (i.e., Sn^4+^/(Sn^4+^ + Sn^2+^)) decreased from ≈71% in the control to 25% with Sn powder addition, and further to ≈7% with OCOS treatment (Figure 2f).

The mechanisms underlying the improvements differ significantly between the two approaches. Sn metals reduce Sn^2+^ oxidation through a sequential process:^[^ ^22^ ^]^ initial oxidation of Sn^2+^, followed by reduction of Sn^4+^ to Sn^2+^, and the consequential generation of additional Sn^2+^. Meanwhile, the OCOS strategy directly inhibits the oxidation of Sn^2+^ to Sn^4+^ via the formation of EtO–Sn complexes, facilitated by lone pair electrons donated by oxygen atoms in ethoxide (EtO^−^) (Figure 1b). These XPS analysis results suggest that the OCOS strategy is more effective than conventional Sn metal addition in preventing the Sn^2+^ oxidation, potentially leading to enhanced LED efficiency.

Furthermore, we performed large‐scale density functional theory (DFT) calculations on a single‐layer 2D (TEA)_2_SnI_4_ perovskite model to elucidate the origin of the enhanced stability (Figure 2g; see Experimental Section and Figure S15, Supporting Information).^[^ ^34^, ^35^ ^]^ Substitution of an iodide site with EtO^−^ yielded a substantial energetic gain of −1.96 eV, highlighting the enhanced energetic stability of the EtO^−^‐coordinated perovskite structure. Projected density of states (PDOS) analysis reveals that this stabilization stems from the markedly stronger coupling between the O‐2p orbitals of EtO^−^ and the Sn‐5s orbitals, compared with the native interaction between I‐5p orbitals and Sn‐5s orbitals. It can be evidenced by the pronounced features in the −8.0 to −5.0 eV range of the PDOS (Figure 2h). This stronger bonding between the O‐2p of EtO^−^ and the Sn‐5s orbitals can reduce iodide vacancies, thereby suppressing the oxidation of Sn^2+^. Beyond this contribution, additional lattice stabilization is achieved through strong (TEA)N‐H ··· O hydrogen‐bonding interactions (Figure S16, Supporting Information).

### 2.3 Effect of OCOS Strategy on the Optical and Structural Properties

**Figure 3:**
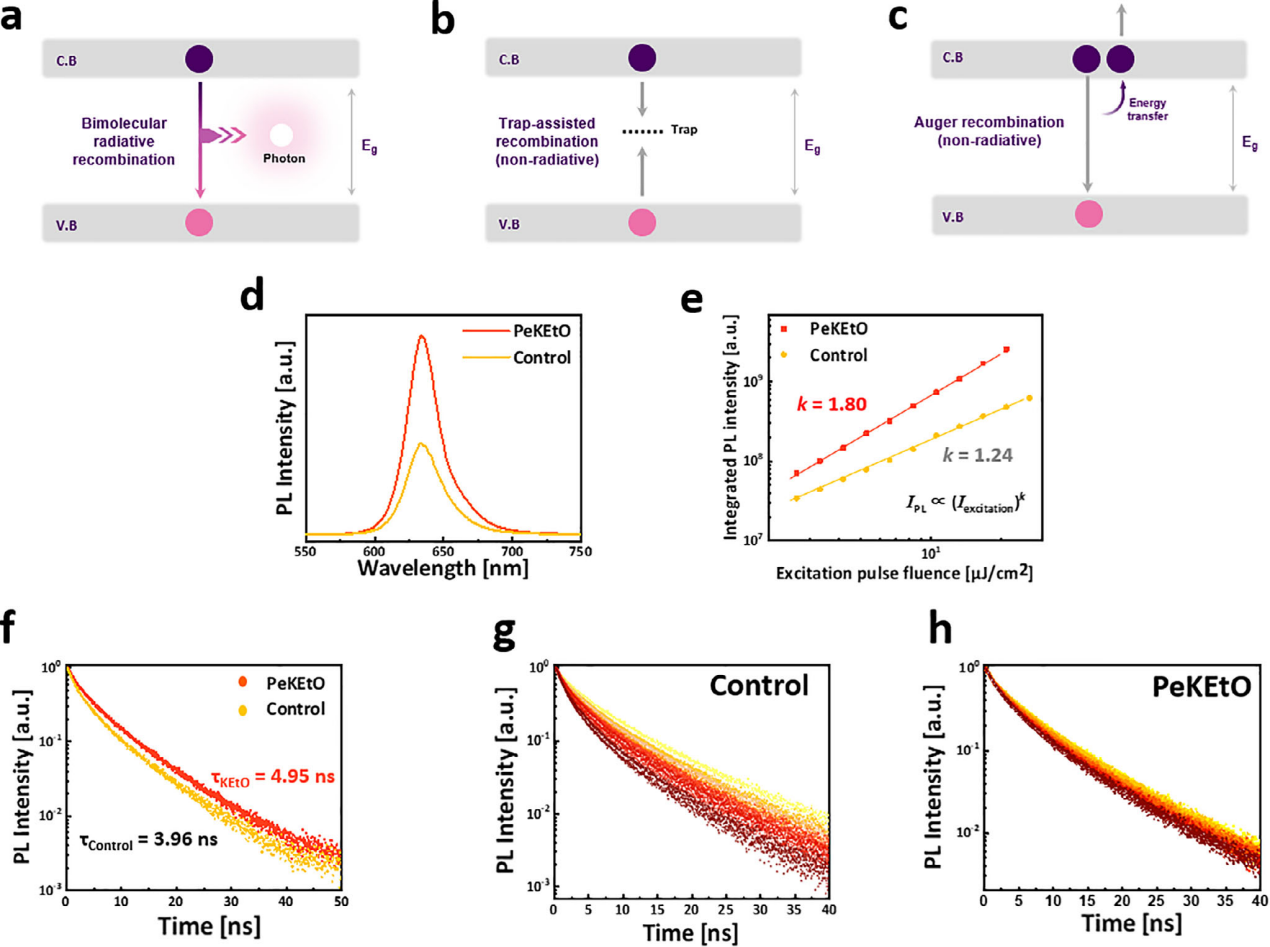
a–c) Schematic diagram of bimolecular radiative recombination, trap‐assisted non‐radiative recombination, and Auger recombination. d) Photoluminescence (PL) intensity comparison between the PeKEtO and control film. e) Power‐dependent PL‐derived *k* values, suggesting increased radiative bimolecular recombination (*k*_control_ = 1.24, *k*_KEtO_ = 1.80). f) Time‐resolved PL (TRPL) decay curves showing carrier lifetimes for the control and PeKEtO films. g,h) Power‐dependent TRPL decay curves for control and PeKEtO films.

The photophysical properties (**Figure** **3**a–c) of the control and PeKEtO films were analyzed to understand the carrier dynamics.^[^ ^19^, ^30^ ^]^ Figure 3d illustrates that the PL intensity of the PeKEtO was higher than that of the control film under identical excitation light power, indicating a reduction in non‐radiative processes. This improvement is attributed to defect passivation facilitated by the alkali metal and organic anion introduced through the OCOS strategy.

To further assess radiative recombination, particularly bimolecular recombination (Figure 3a), power‐dependent photoluminescence (PDPL) analysis was conducted by varying the excitation pulse laser power (Figure S4, Supporting Information). The parameter *k* represents the exponential relationship between excitation pulse power and PL intensity, where a *k* value approaching 2 indicates enhanced bimolecular radiative recombination involving two charge carriers.^[^ ^36^, ^37^ ^]^ For the control film, *k* was ≈1.24, whereas it increased significantly to ≈1.80 with the incorporation of K‐EtO (Figure 3e), demonstrating improved bimolecular recombination efficiency.

Time‐resolved photoluminescence (TRPL) results further confirmed improved charge carrier dynamics, showing an increase in the average carrier lifetime from 3.92 ns (control) to 4.95 ns (PeKEtO) (Figure 3f). This enhancement in PL lifetime is attributed to reduced trap‐assisted non‐radiative recombination (Figure 3b), achieved through the strategic passivation by the alkali metal (K^+^) and organic anion (EtO^−^). Additionally, Figure S5 (Supporting Information) presents additional TRPL data for films incorporating another alkali metal, such as Li^+^, Na^+^, Cs^+,^ with EtO^−^ (namely, PeLiEtO, PeNaEtO, PeCsEtO), demonstrating a notable lifetime enhancement compared to the control film. The PeCsEtO film, containing *n* ≥ 2 phases, exhibited a longer carrier lifetime, which is influenced by the number of octahedral layers (*n*‐value) and a widened quantum‐well modulated by the organic layer (TEAI).^[^ ^31^, ^38^ ^]^ Phases with *n* ≥ 2 show reduced exciton self‐trapping states and lower electron‐phonon interactions, thereby extending carrier lifetimes. Nevertheless, PeCsEtO is unsuitable for LED applications due to the EL double peaks undesirable feature for high color purity. This issue will be further addressed in the device performance section.

A power‐dependent TRPL (PD‐TRPL) analysis was conducted by modulating the excitation laser pulse power to investigate carrier dynamics under varying excitation conditions. As the excitation power increased, the excited carrier population in the conduction band rose correspondingly, leading to increased three‐body Auger recombination (Figure 3c). Since Auger recombination occurs much faster than ideal bimolecular recombination, a noticeable reduction in carrier lifetime with increasing power indicates intensified Auger recombination. Figure 3g,h shows that the PeKEtO film exhibited a considerably smaller decrease in lifetime compared to the control film, suggesting that the incorporation of KEtO into the tin‐perovskite significantly suppressed Auger recombination. The organic anion (EtO^−^) plays a primary role in directly suppressing oxidation and passivating defects,^[^ ^23^, ^26^ ^]^ while the alkali metal (K^+^) further stabilizes the tin‐perovskite structure.^[^ ^23^, ^24^, ^31^ ^]^ Previous studies have indicated that surface defects trap carriers, creating localized high‐density regions and increasing the likelihood of Auger recombination.^[^ ^39^, ^40^ ^]^ Additionally, atomic force microscope (AFM) measurements (Figure S6, Supporting Information) revealed that the OCOS strategy significantly reduced surface roughness from 18.8 to 9.73 nm. This enhanced thin‐film morphology facilitates more efficient charge carrier transport, minimizes trap states at interfaces, reducing non‐radiative recombination and thereby increasing radiative recombination.

### 2.4 Performance Evaluation of Tin‐Perovskite PeLEDs Incorporating OCOS Strategy

**Figure 4:**
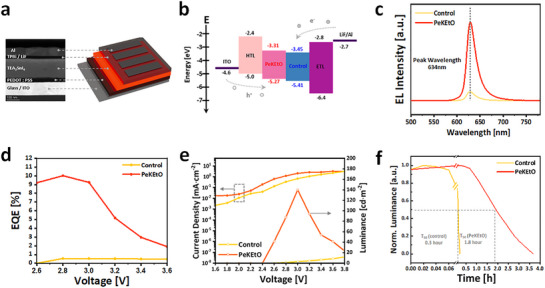
a) Schematic and cross‐sectional transmission electron microscopy (TEM) image of the tin‐perovskite LED structure. b) Energy band diagram of reference and PeKEtO‐based PeLED devices. c) Electroluminescence (EL) spectra of control and PeKEtO showing a full width half maximum (FWHM) under 30 nm, satisfying Rec.2020 standards. d) EQE‐Voltage curves comparing control and PeKEtO PeLEDs. e) *J–V* and *L–V* curves for PeLEDs based on control and PeKEtO. f) Operational lifetime (T_50_) comparison of PeLEDs constructed from control and PeKEtO films.

We evaluated the effectiveness of the OCOS strategy on the performance of LED devices with the structure: indium tin oxide (ITO) / poly(3,4‐ethylenedioxythiophene) polystyrene sulfonate (PEDOT:PSS) / (TEA)_2_SnI_4_ perovskite / 1,3,5‐Tris(1‐phenyl‐1Hbenzimidazol‐2‐yl)benzene (TPBi) / lithium fluoride (LiF) /Al, as depicted in **Figure** **4**a. A cross‐sectional transmission electron microscope (TEM) image of the PeLEDs is presented in Figure S7 (Supporting Information). Scanning electron microscope (SEM) and energy‐dispersive X‐ray spectroscopy (EDS) mapping confirm that the OCOS strategy effectively improves the overall film morphological crystalline quality with a reduced density of pinholes and ensures uniform elemental distribution (Figure S8, Supporting Information). The improved crystallinity is further supported by the increased peak intensity in XRD (Figure S3, Supporting Information). Figure 4b provides a schematic representation of the energy band alignment and charge transport pathways in the device, derived from ultraviolet photoelectron spectroscopy (UPS, detailed in Figure S9, Supporting Information).

As shown in Figure 4c, application of the OCOS strategy, specifically with PeKEtO, markedly improved the EL intensity of the PeLEDs compared to the control. The FWHM remained below 30 nm, demonstrating superior color purity and full compliance with Rec.2020 standards. Although the PeCsEtO film exhibited the longest carrier lifetime (Figure S5, Supporting Information), its EL spectrum showed a distinct double peak at 629 and 698 nm under higher injection voltage (Figure S10, Supporting Information). This suggests that Cs^+^ is less suitable for next‐generation LEDs targeting Rec.2020 standards due to its inability to achieve single‐peak emission with high color purity.

Figures S11 and S12 show the performance changes by adjusting the thicknesses of the emission layer (EML), electron transporting layer (ETL), hole transporting layer (HTL), and lithium fluoride (LiF) layer. Optimizing layer thickness is crucial, as excessively thin layers can suffer from quenching at the cathode electrode and poor light extraction,^[^ ^41^, ^42^ ^]^ while an optimal thickness help reduce spatial trap state density and improve charge confinement, improving light outcoupling.^[^ ^43^, ^44^ ^]^ EML and HTL thicknesses were optimized by adjusting (TEA)_2_SnI_4_ and PEDOT:PSS precursor solution concentrations, while ETL and LiF thicknesses were tuned through physical vapor deposition. PeLEDs with 140 nm EML, 24 nm HTL, 40 nm ETL, and 1 nm LiF showed balanced EQE and luminance improvement, achieving a well‐optimized structure for efficient charge transport and radiative recombination.

The PeKEtO‐treated device demonstrated significant performance improvements compared to the control, with EQE increasing from 0.54% to 10.01%, and luminance from 11.85 to 139.9 cd m*^−^* ^2^ (Figure 4d–f). It can be attributed to reduced trap density, as confirmed by space‐charge‐limited current (SCLC) measurements. Figure S14 (Supporting Information) presents the SCLC results from hole‐only devices (ITO/PEDOT:PSS/emissive layer/10 nm MoO_3_/ 120 nm Ag), revealing a decreased trap‐filled limit voltage (V_TFL_) from 3.41 to 2.69 V, and a reduced trap density from 5.48 × 10^16^ to 4.32 × 10^16^ cm^−3^ in the PeKEtO device. Additionally, the enhanced performance can be attributed to improved band alignment (Figure 4b). The PeKEtO film exhibited shallower valence band energy level (−5.27 eV) than the control (−5.41 eV), resulting in more effective hole injection from the hole‐transporting layer. Trends in device performance for other alkali‐metals‐treated samples (e.g., PeLiEtO, PeNaEtO) are provided in Figure S13 (Supporting Information). Among them, PeKEtO delivered the highest overall performance in terms of FWHM, luminance, and EQE. PeLEDs with KEtO demonstrated enhanced charge injection and radiative recombination, achieving high luminance at low current densities, and its narrow FWHM, indicative of improved structural and thermal stability, contributed to improved long‐term stability. Operational lifetime (T_50_) measurements revealed that the PeKEtO PeLED achieved a T_50_ of 1.8 h, compared to only 0.3 h for the control devices at an initial luminance of 30 cd m*^−^* ^2^ under constant current (Figure 4f). Degradation of PeLEDs during operation is typically caused by ion migration, charge‐induced oxidation, and exposure to moisture and oxygen.^[^ ^45^, ^46^ ^]^ Therefore, the enhanced operational stability of PeKEtO devices compared to the control device can be attributed to the effectively suppressed oxidation of tin perovskite by the coordination of KEtO with the perovskite lattice.

## 3 Conclusion

This study presents a novel organic anion‐coordination for oxidation suppression (OCOS) strategy to enhance the performance and stability of tin‐based perovskite light‐emitting diodes. By incorporating alkali metal–organic anions into the perovskite structure, the OCOS approach effectively suppressed the oxidation of Sn^2+^ to Sn^4+^, a major degradation pathway in tin‐based perovskites. This approach facilitated the formation of stable Sn^2+^ complexes, significantly reducing Sn^4+^ content in the perovskite films. Density functional theory (DFT) calculations were employed to quantify the energetic stabilization of 2D (TEA)_2_SnI_4_ induced by the substitution of I^−^ with EtO^−^ and to explain its electronic structural origins. This suppression of oxidation not only improved the photoluminescence intensity but also prolonged carrier lifetime, indicating enhanced radiative recombination and reduced non‐radiative losses. The incorporation of potassium ethoxide (K‐EtO), in the perovskite films led to remarkable performance improvements, increasing EQE from 0.54% to 10.01%, and maximum luminance from 11.85 to 139.9 cd m^−2^. Furthermore, the operational lifetime (T_50_) of the devices was extended from 0.3 to 1.8 h. Morphological analysis revealed a substantial reduction in surface roughness from 18.8 to 9.73 nm, indicative of improved thin‐film quality and reduced trap states. These findings highlight the potential of the OCOS strategy to overcome critical challenges in tin‐based perovskites, paving the way for the development of efficient and stable lead‐free PeLEDs.

## 4 Experimental Section

### Materials

Tin(II) iodide (SnI_2_; 99.99%), N,N‐dimethylformamide (DMF, 99.8%), dimethylsulfoxide (DMSO, 99.9%), lithium fluoride (LiF, 99.99%), toluene, and ethoxide with various alkali metals were purchased from Sigma–Aldrich. 2‐thiophenethylammonium iodide (TEAI) was purchased from Greatcell Solar Korea. 2,2′,2′’‐benzene‐1,3,5‐triyltris(1‐phenyl‐1H‐benzimidazole) (TPBi) was purchased from Luminescence Technology Corp. Glass with indium tin oxide (ITO) substrates were purchased from AMG Glass Tech. Poly(3,4‐ethylenedioxythiophene)/poly(styrenesulphonate) (PEDOT:PSS) aqueous solutions were purchased from Heraeus, Germany. All materials were used as received without further purification.

### Preparation of Perovskite Precursor

TEAI and SnI_2_ were mixed in anhydrous DMF:DMSO (4:1) solvent at a concentration of 0.3 m maintaining a stoichiometric ratio of TEAI:SnI_2_ = 2:1. For PeKEtO and other solutions treated with the OCOS strategy, the stoichiometric ratio was adjusted to TEAI:SnI_2_:potassium ethoxide (KEtO) = 2:1:0.1. The precursor solutions were stirred overnight at 300 rpm and room temperature using a stirring bar inside an N_2_‐filled glovebox, where both H_2_O and O_2_ concentrations were maintained below 0.1 ppm.

### Device Fabrication

ITO‐coated glass substrates were cleaned ultrasonically in acetone and isopropyl alcohol for 30 min each, followed by drying in an oven at 120 °C for 10 min. The cleaned substrates were treated with O_2_ plasma for 10 min to make the surfaces hydrophilic. PEDOT:PSS was spin‐coated onto the treated ITO substrates in two steps: 500 rpm for 10s, 3000 rpm for 30s. The spin‐coated substrates were annealed in air on a hotplate at 100 °C for 30 min. After cooling to room temperature, a two‐step process was employed to spin‐coat the tin‐perovskite films within a glove box. Initially, the precursor solution (0.3 m) was applied to the PEDOT:PSS‐coated substrate and then spin‐coated at 500 rpm for 5s, and 4000 rpm for 30s. During the second step, 300 µL of toluene solution was sprayed onto the substrates 10s after the spin‐coating started. The coated substrates were baked at 100 °C for 10 min. The prepared substrates were vacuum‐packaged and then transferred to a high‐vacuum thermal evaporator. Sequential layers of TPBi (≈25 nm), LiF (≈1 nm), and Al (≈60 nm) were deposited using a shadow mask, under a pressure below 10^−7^ torr.

### Characterization

The proportions of Sn^2+^ and Sn^4+^ were analyzed by XPS using a Thermo VG scientific K‐alpha instrument. Cross‐sectional samples were prepared using a focused ion beam (FIB) on a Helios 450 system and analyzed by transmission electron microscopy (TEM) using a JEOL JEM‐ARM200F instrument. PL characteristics of the samples were analyzed using a PL spectrometer (LabRAM HR Evolution Visible NIR, HORIBA). Crystal structures were examined by a high‐resolution thin‐film X‐ray diffractometer (XRD) using a RIGAKU D/MAX 2500 instrument. The optical and electrical properties of the PeLEDs were investigated by measuring EL spectra and current‐voltage‐luminance characteristics using a spectroradiometer (CS‐2000, Konika Minolta) and programmable source meter (Keithley model 2400).

### DFT Calculations

DFT calculations were performed using the VASP package based on the projected augmented wave method.^[^ ^47^ ^]^ Many‐electron exchange‐correlation interactions were described within the Perdew–Burke–Ernzerhof form of generalized gradient approximation augmented by Grimme's DFT‐D3 method.^[^ ^48^ ^]^ The kinetic energy cut‐off for the plane wave basis was set at 400 eV. The atomic structures were optimized until the total energy and the Hellmann–Feynman forces on each atom reached the 10^−4^ eV and 0.01 eV Å^−1^ levels, respectively. More details can be found in the References.^[^ ^34^, ^35^ ^]^

Single‐layer 2D (TEA)_2_SnI_4_ crystal models have been prepared in a fashion similar to that used for the identification of self‐assembled monolayer structures.^[^ ^49^, ^50^ ^]^Three supercells comprised of 2 × 2, 2 × 4, and 4 × 4 unit cells were first considered, and atomic structures were relaxed using the 4 × 4, 4 × 2, and 2 × 2 Monkhorst‐Pack k‐point samplings, respectively (Figure S15, Supporting Information). It was then found that the minimum‐energy TEA packing ordering assumes a 2 × 2 unit‐cell superstructure in which the–CH_2_–NH_3_^+^ head groups and thiophene tail groups of surface TEA adopted zigzag‐like and parallel configurations, respectively, as shown in Figure S16 (Supporting Information). Referring to this supercell as (TEA)_2_SnI_4_ and the corresponding model in which an I^−^ is replaced by EtO^−^ as PeEtO, the substitutional reaction was then considered

(1) $${\left(\mathrm{TEA}\right)}_{2}\mathrm{Sn}I_{4}+\mathrm{Et}O^{-}\;\to \mathrm{PeEtO}+\;I^{-}$$

and found that EtO^−^ binds to the Sn cation stronger than I^−^ by −1.96 eV. For subsequent PDOS electronic structure analyses, 12 × 12 × 1 k‐points were sampled.

## Conflict of Interest

The authors declare no conflict of interest.

## Author Contributions

S.L. and H.L. contributed equally to this work. S.L., H.L., and J.L. conceived and designed the experiments and prepared the manuscript. H.L. fabricated and measured the LED devices. S.L., H.L., C.K., J.L., and S.B.L. performed analyses P.L., TRPL. J.K., S.Y.L., and H.K. analyzed XRD and TEM. S.L., H.L., and Y.L. performed XPS analysis. S.J. and W.Y. revised the manuscript. All authors discussed the results and commented on the manuscript. J.P., H.Y., and R.‐G.L. performed DFT calculations and analyzed results together with Y.‐H.K. Y.‐H.K. oversaw the theoretical part of the project and revised the manuscript based on the input from J.P. and H.Y.

## Supporting information



Supporting Information

## Data Availability

The data that support the findings of this study are available in the supplementary material of this article.
