# Supplementary material for: Lead‐Free Tin‐Based Perovskite LEDs Toward Rec. 2020: Organic Anion Coordination for Oxidation Suppression
Source: Adv Sci (Weinh). 2025 Sep 16;13(2):e11006. doi: 10.1002/advs.202511006 (PMC12786335; doi:10.1002/advs.202511006)
Supplement: Supplementary file 1 — Supporting Information [file ADVS-13-e11006-s001.docx]

Supporting Information

**Lead-free tin-based perovskite LEDs towards Rec. 2020:Organic anion coordination for oxidation suppression**

*Seungjae Lee, Heeseung Lee, Joonho Park, Hyeonwoo Yeo,* *Junho Kim, Changjo Kim, Hyojun Kim, Seyun Lee, Jihyung Lee, Yun Hoo Kim, Seungbok Lee, Seonju Jeong,* *Wu Bin Ying,* *Ryong-Gyu Lee,* *Yong-Hoon Kim, and Jung-Yong Lee**


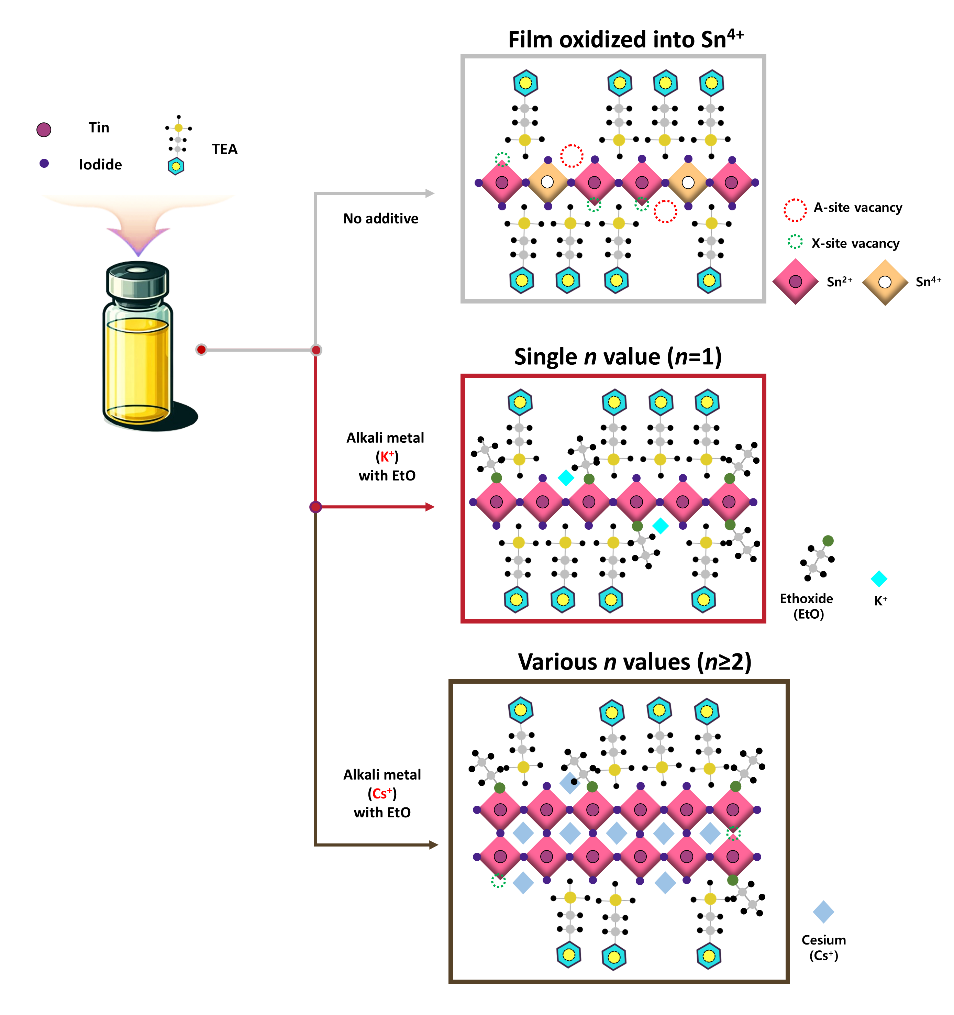


**Figure S1.** Schematic illustrations of perovskite structures with and without OCOS additives

The topmost schematic in Figure S1 represents a pristine 2D tin-perovskite (TEA)_2_SnI_4_, which contains A-site cation vacancies, X-site halide vacancies, and oxidized tin (Sn^4+^). The second schematic illustrates a tin-perovskite with K^+^ - ethoxide (EtO^-^) additives, where K⁺ ions passivate A-site vacancies, and EtO⁻ anions passivate X-site halide vacancies. Additionally, EtO^-^ suppresses the oxidation Sn^2+^ to Sn^4+^, improving the redox stability. The bottom schematic depicts a tin-perovskite film treated with Cs^+^-EtO^-^, where the larger Cs^+^ ions induce higher-order (*n*≥2) perovskite phases within the lattice and at grain boundaries.


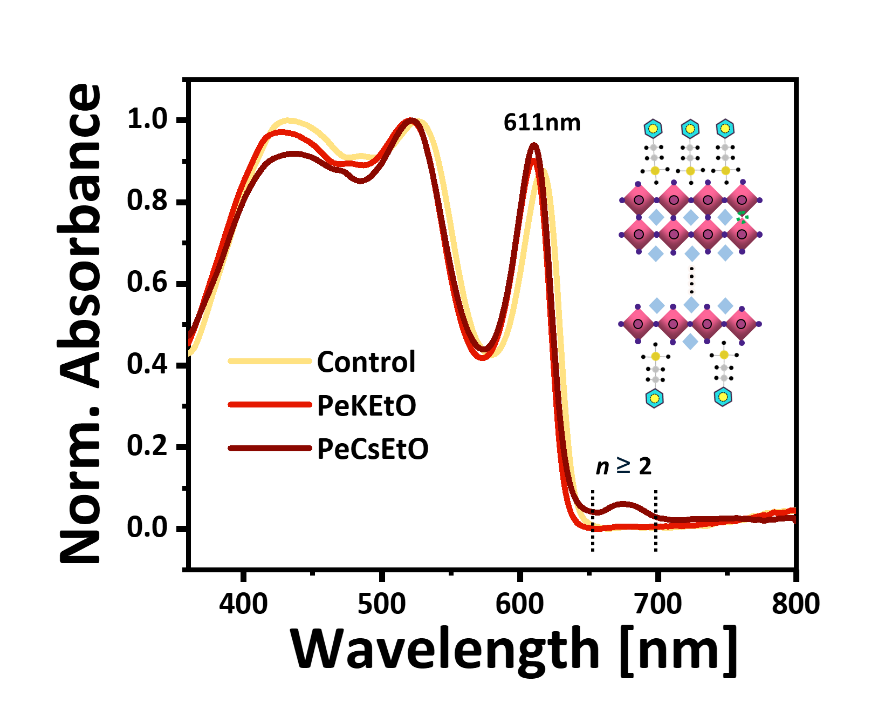


**Figure S2**. Ultraviolet-visible (UV-Vis) absorption spectra of control, PeKEtO, and PeCsEtO films

The PeKEtO films maintain absorption peaks at 431, 523, and 611 nm, nearly identical to the untreated sample, indicating retention of a single octahedral layer phase (*n* = 1), consistent with the pristine 2D structure (Figure S2). Meanwhile, PeCsEtO absorption spectra exhibits an additional peak around 680 nm, suggesting the presence of multi-octahedral layer phase (*n* ≥ 2) phases.


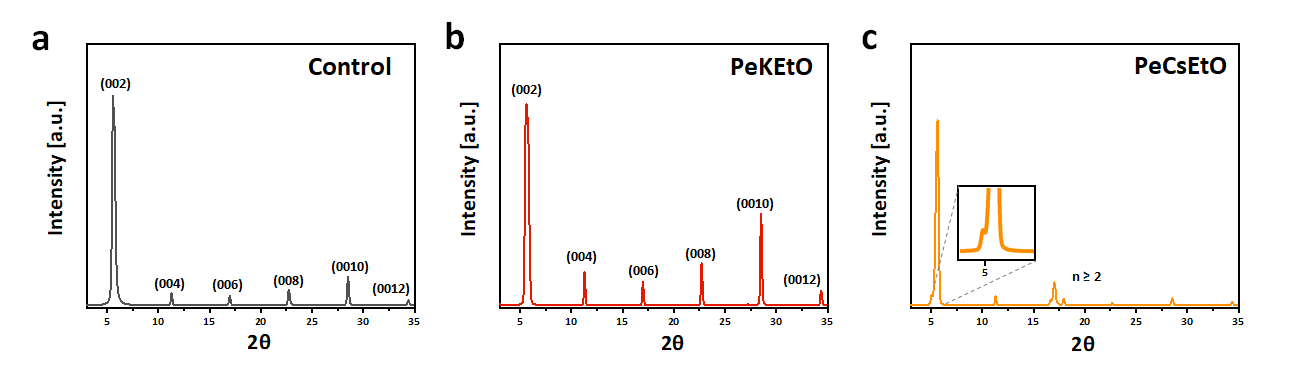


**Figure S3**. X-ray diffraction (XRD) spectra of (a) control, (b) PeKEtO, and (c) PeCsEtO films.

The control and PeKEtO films display multiple sharp diffraction peaks characteristic of conventional 2D perovskites. Notably, the PeKEtO displays enhanced intensity in high-order (00n ; n=4,6,8,10,12) sub-peaks, indicating improved crystallinity and phase purity compared to the control. In contrast, the PeCsEtO film presents a main (002) peak near 5.6°, which appears to be slightly broadened and partially split shoulder peak as shown in Figure S3c. The full width half maximum (FWHM) of the (002) peak increases from 0.29° (control) to 0.38°, demonstrates increased crystal dimensionality. As the *n* value increases, the number of inorganic layers (SnI_6_ octahedra) per repeating unit grows, resulting in thicker repeating units and larger *d*-spacing. According to Bragg’s law, an increase in *d*-spacing leads to a lower diffraction angle. Thus, the structural changes in PeCsEtO reduce the diffraction angle, causing an additional peak at 4.9°, branching from the main peak at 5.6°. Furthermore, an additional diffraction peak appears in the 15°-20° range for PeCsEtO, consistent with previous reports, providing additional evidence of the increased dimensionality in the perovskite structure.


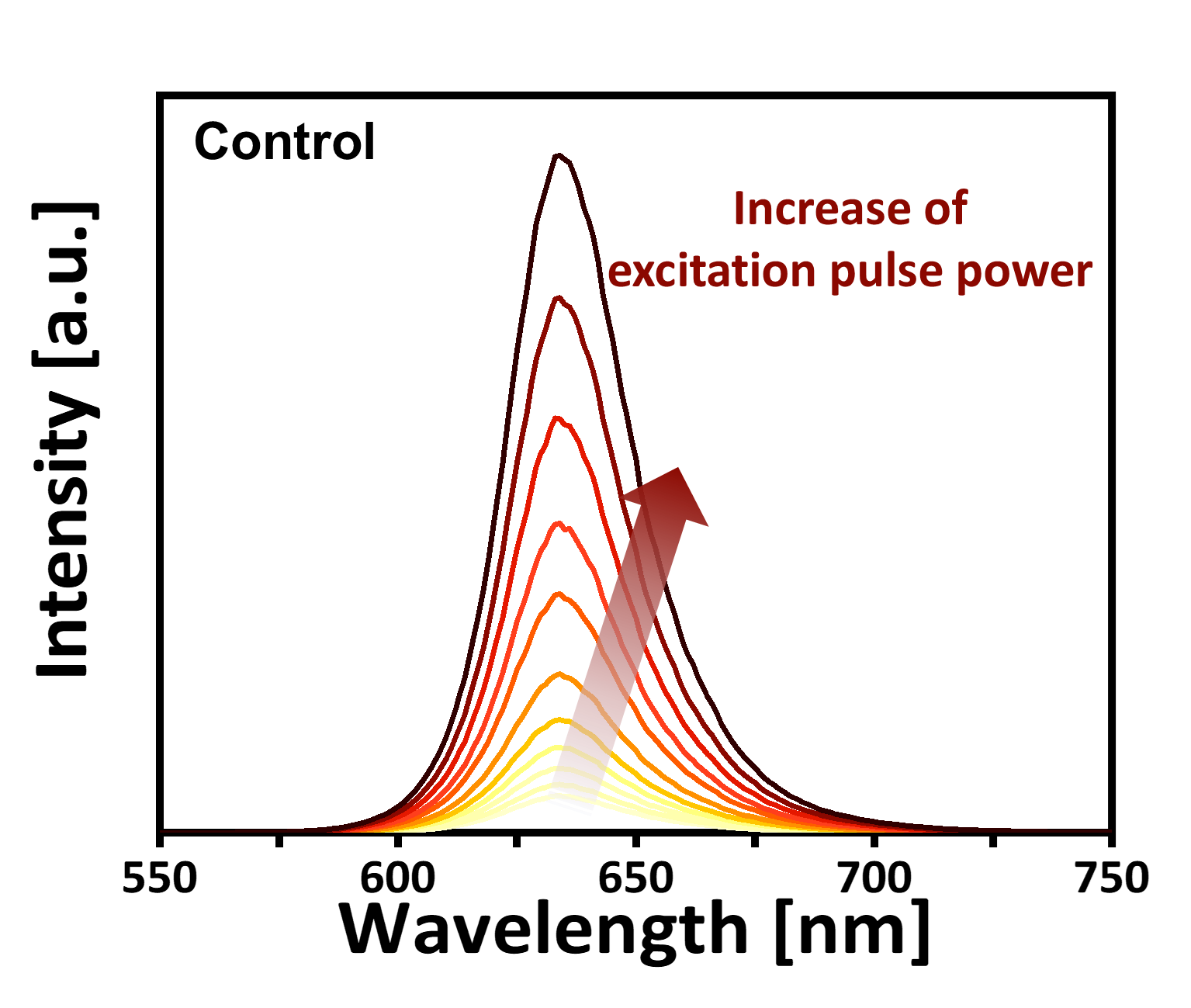


**Figure S4.** Excitation pulse power incrementally applied for power-dependent photoluminescence.

A gradual increase in excitation pulse power was implemented for power-dependent photoluminescence measurements, and this method was consistently applied to both the control and PeKEtO-treated thin films.


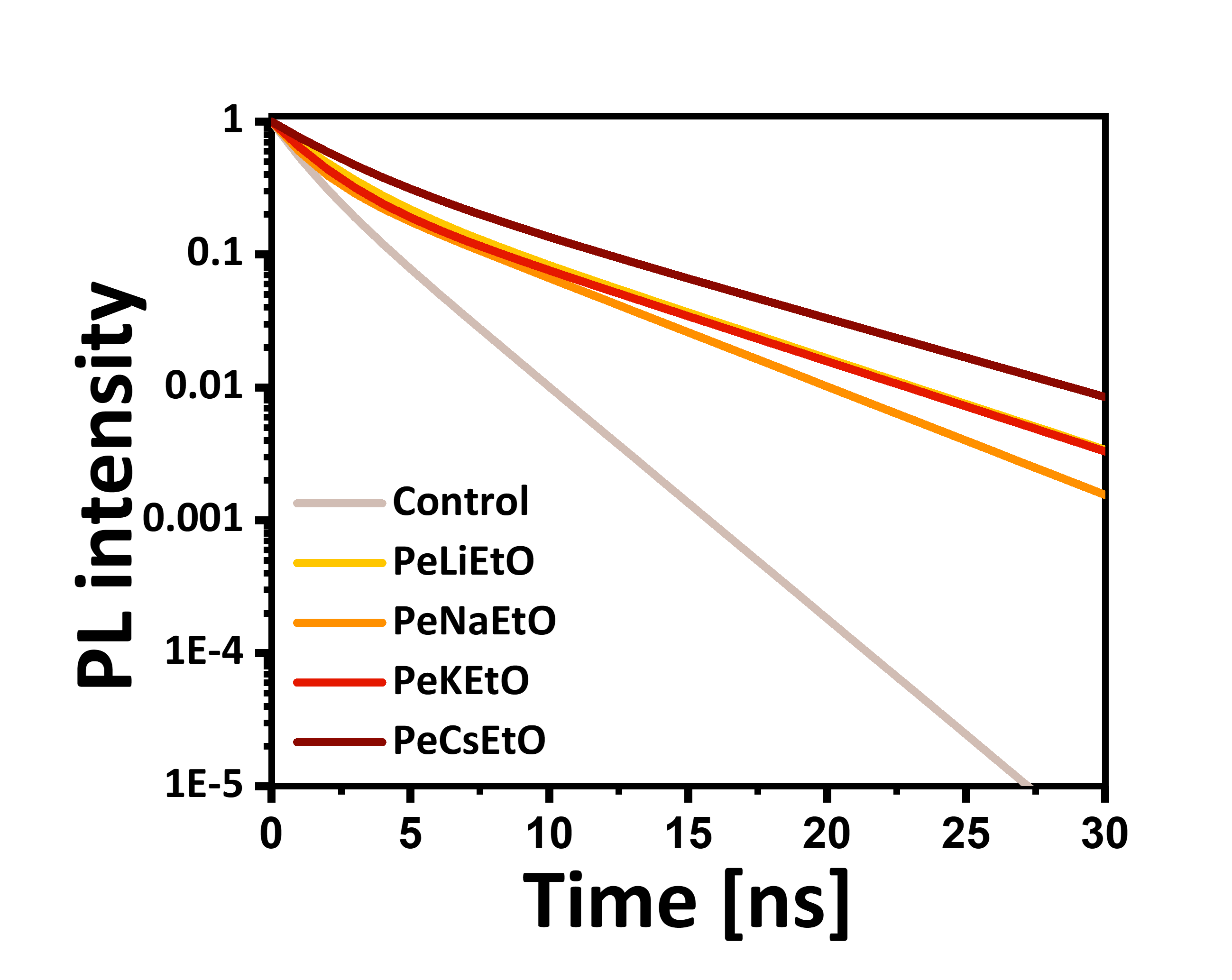


**Figure S5**. Time-resolved photoluminescence (TRPL) spectra of films treated with various alkali metals

The OCOS strategy significantly enhances the carrier lifetime in tin-perovskite films (Figure S5). In the cases of PeLiEtO, PeNaEtO, and PeKEtO, their carrier lifetimes are clearly improved compared to the control. Specifically, PeCsEtO films exhibit improved lifetimes due to the formation of higher-order (*n* ≥ 2) phases. However, these films are unsuitable for light-emitting diode (LED) applications due to the electroluminescence (EL) double peaks (Figure S10).


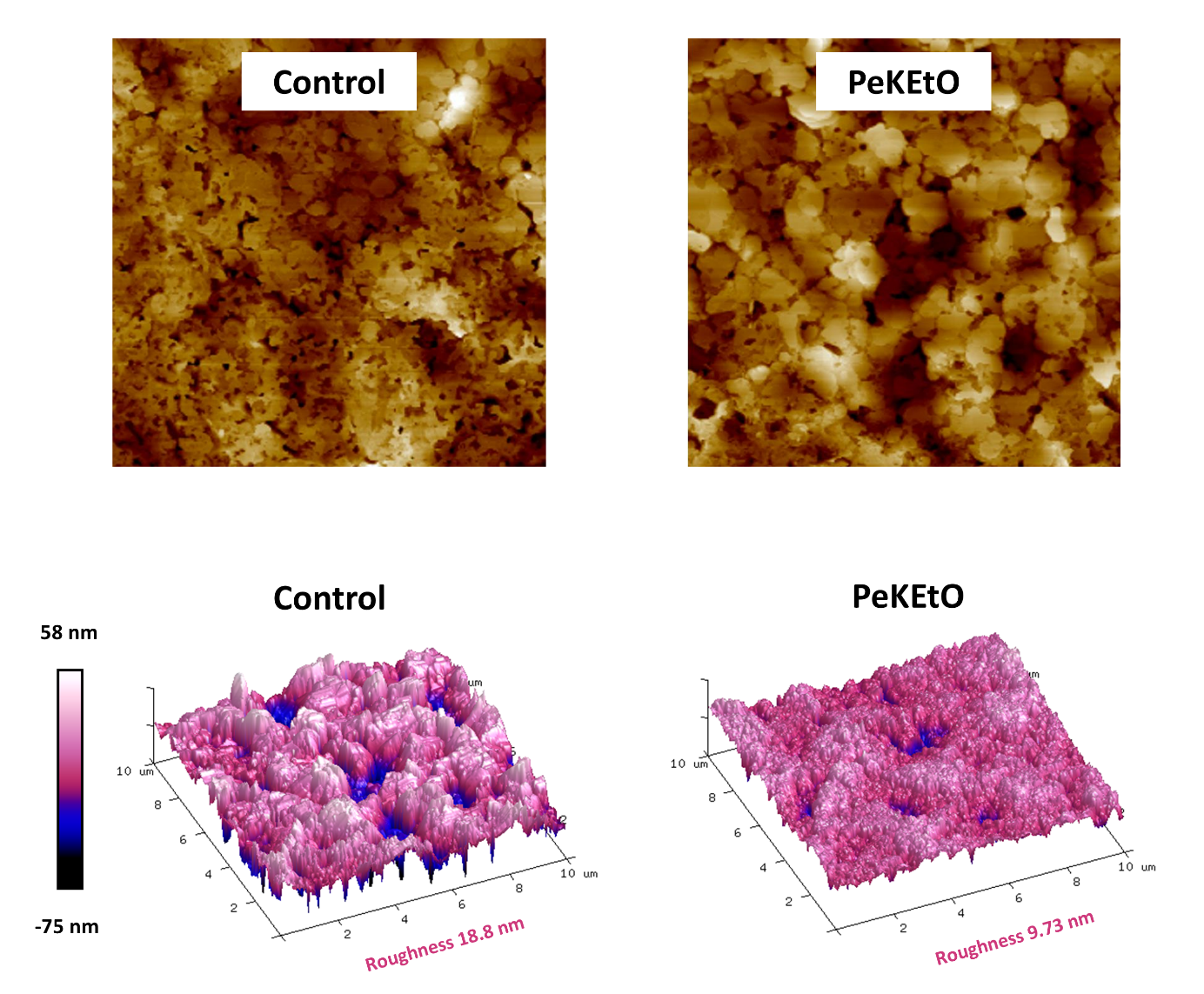


**Figure S6**. Atomic force microscopy (AFM) images illustrating the surface morphology and roughness of control and PeKEtO films

Atomic force microscopy (AFM) analysis reveals that KEtO treatment reduces surface roughness from 18.8 nm to 9.73 nm, resulting in smoother morphology that improves charge transport, minimizes trap states, and reduces non-radiative recombination, thereby enhancing PeLED performance and longevity.


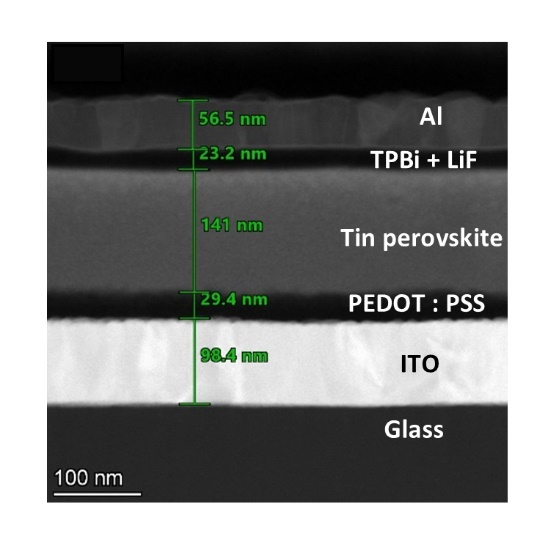


**Figure S7**. Cross-sectional transmission electron microscopy (TEM) image of the (TEA)_2_SnI_4_ perovskite PeLED

The cross-sectional TEM image of the tin perovskite LED reveals the distinct layer structure of the device (Figure S7).


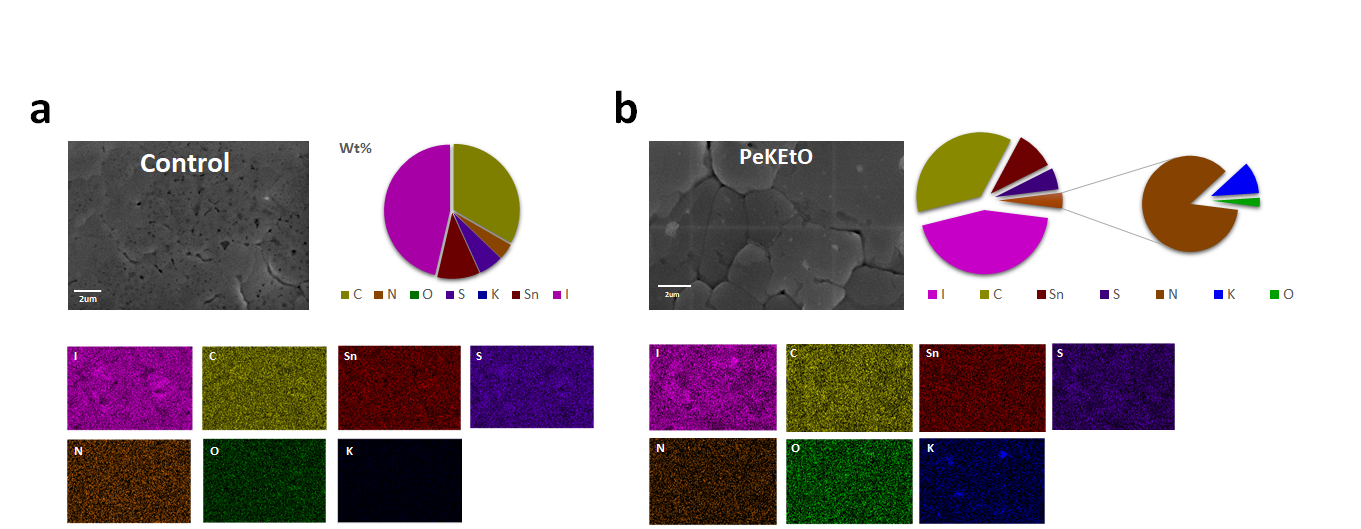


**Figure S8.** Scanning electron microscopy (SEM) and energy-dispersive X-ray spectroscopy (EDS) mapping images of (a) control and (b) PeKEtO films.

Figure S8 a and b compares the surface morphology and elemental distribution of perovskite films before and after KEtO addition. Upon incorporation of KEtO (Figure S8b), notable changes in surface morphology are observed, along with the clear detection of potassium and increased oxygen signals by ethoxide relative to control film. These results support the effective integration of the organic anion and its impact on the structural and chemical environment of the film


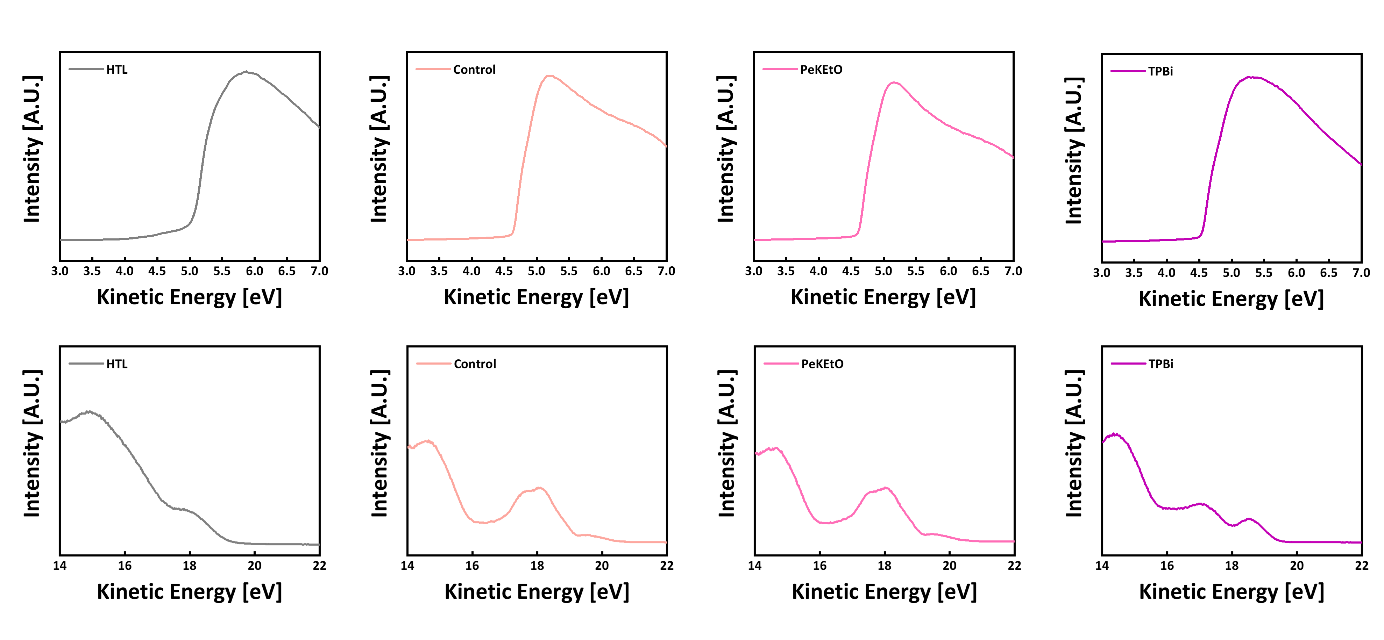


**Figure S9.** Ultraviolet photoelectron spectroscopy (UPS) spectra of the HTL, control, PeKEtO, and 2,2’,2’’-benzene-1,3,5-triyltris(1-phenyl-1H-benzimidazole) (TPBi)

The measured work function values for the HTL, control, PeKEtO, and TPBi are 5.18, 4.64, 4.61, 4.54 eV, respectively (Figure S9).


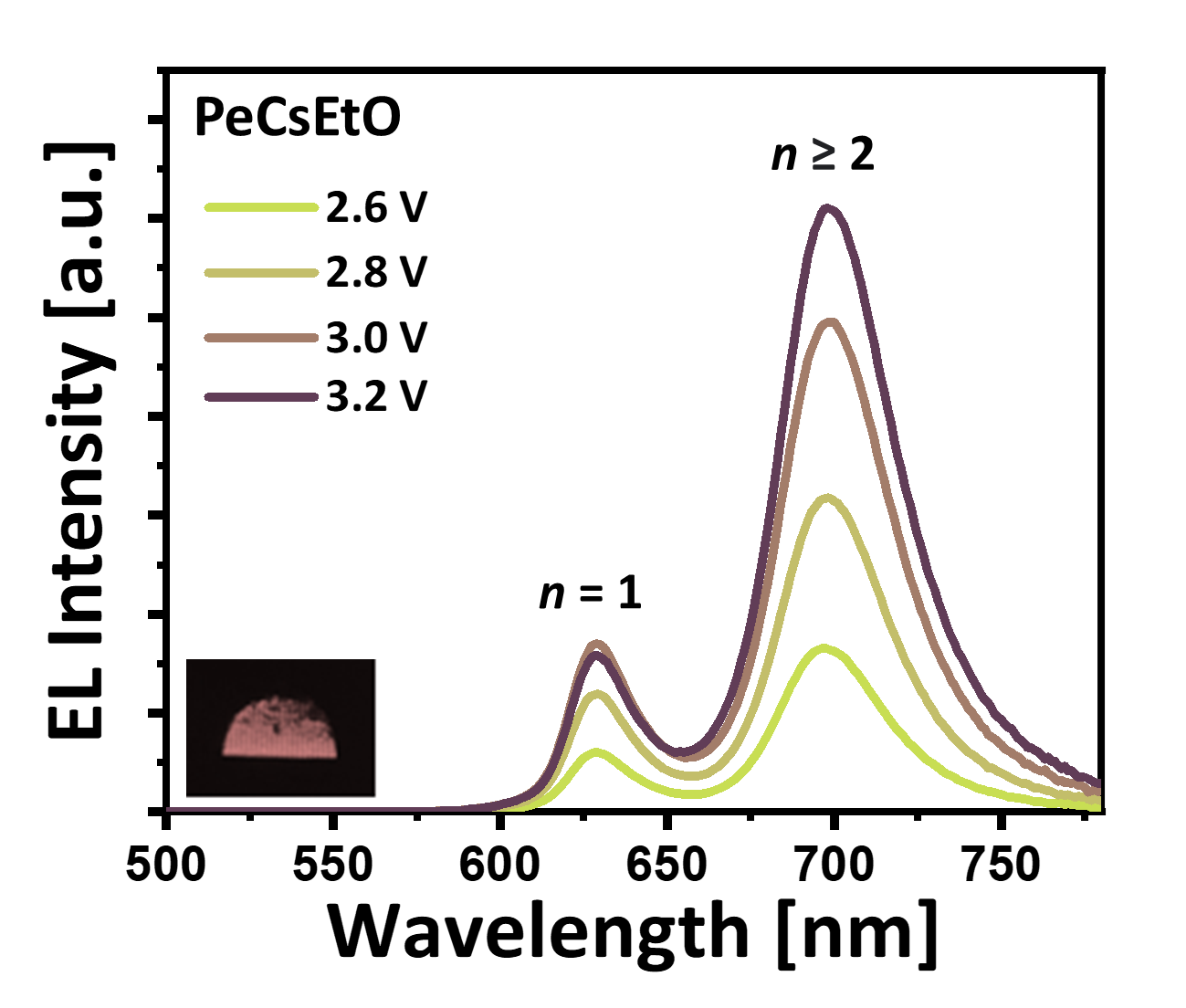


**Figure S10.** Electroluminescence (EL) spectra of a PeLED utilizing PeCsEtO as the emission layer under varying applied bias, showing distinct double peaks at 630 nm and 700 nm

The presence of Cs⁺ induces structural modifications compared to KEtO-treated films, leading to multi-phase (n ≥ 2) formation and resulting in broader, dual-peak emission. Consequently, Cs^+^ as an alkali metal may be unsuitable for next-generation LEDs aiming to satisfy Rec.2020 standards.


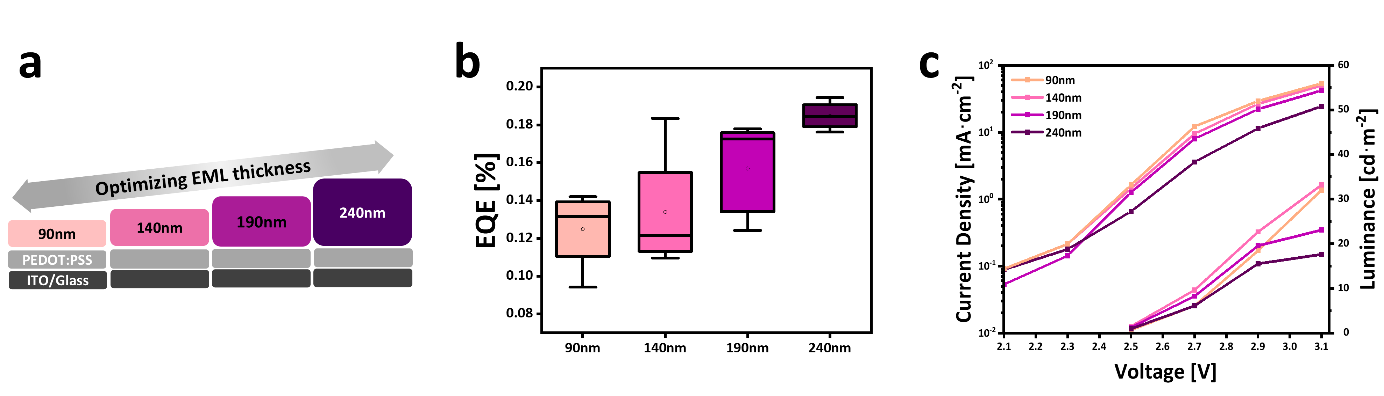


**Figure S11.** Device performance trends related to variations in the emission layer thickness, ranging from 90 nm to 240 nm, using the control (TEA)_2_SnI_4_

Figure S11 shows that increasing the EML thickness from 90 to 240 nm improves EQE from 0.13% to 0.18%, but luminance decreases from 33 cd m^‑2^ to 18 cd m^-2^. Notably, the most substantial decrease in current density occurs at an EML thickness of 240 nm. An EML thickness of 140 nm is selected as optimal, maintaining reasonable luminance and FWHM below 30 nm.


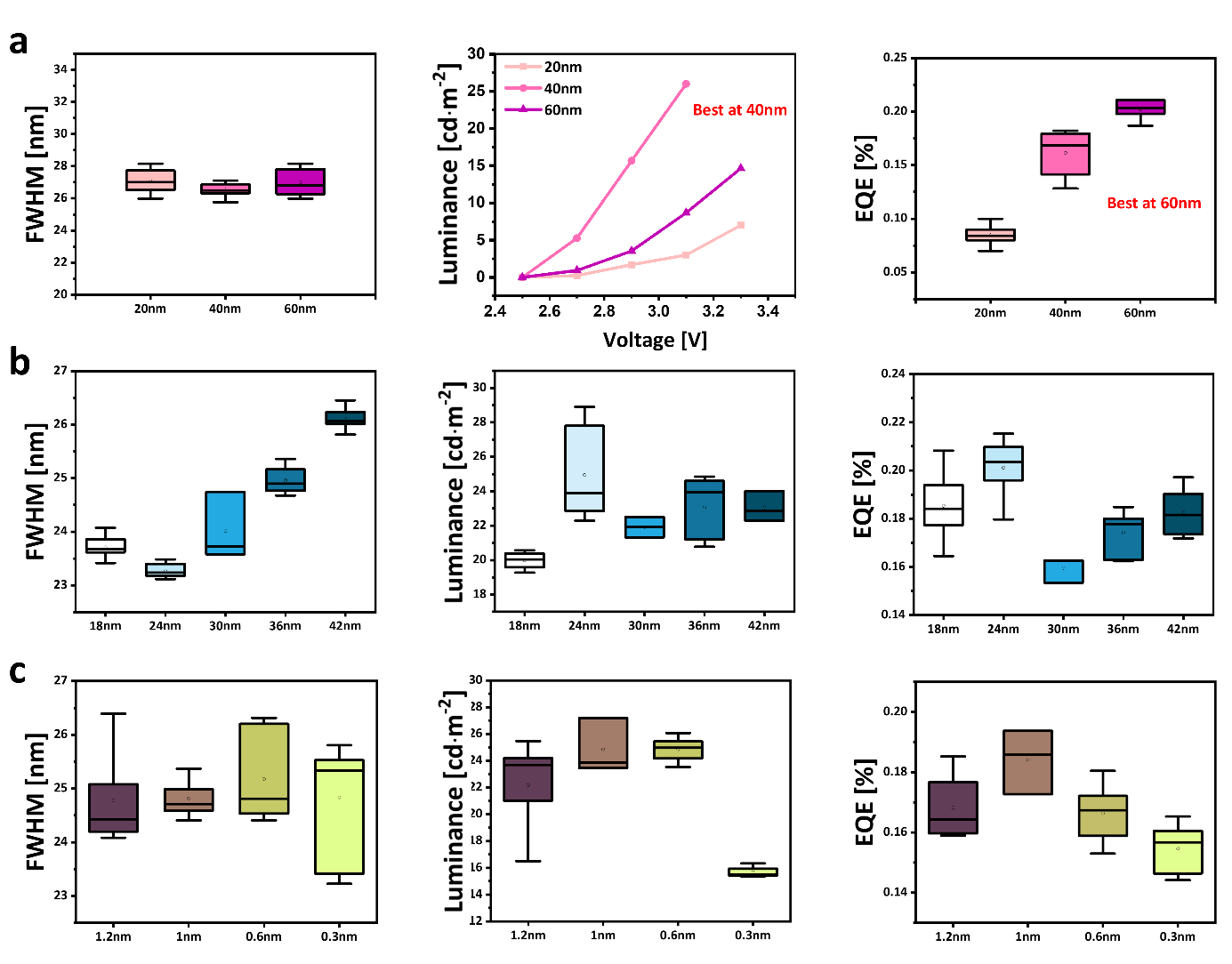


**Figure S12.** Performance of PeLEDs as a function of thickness variations in (a) ETL (TPBi), (b) HTL (PEDOT:PSS), and (c) LiF. The emission layer was fixed as control (TEA)_2_SnI_4_

The ETL thickness is optimized at 40 nm to facilitate electron injection, hole-blocking, and exciton confinement. For the HTL, both luminance and EQE peak at 24 nm. The optimal LiF interlayer thickness is determined to be 1 nm.


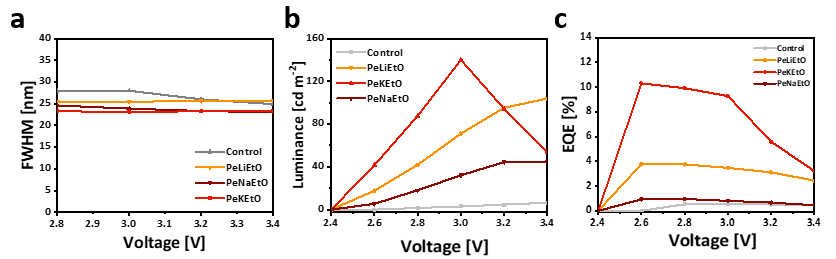


**Figure S13.** Performance trends showing the (a) full width half maximum (FWHM), (b) luminance, and (c) EQE of PeLEDs depending on the alkali metal type

Figure S13 compares the performance of various PeLEDs as a function of applied voltage. As shown in Figure S13a, OCOS-treated devices showed narrower FWHM compared to the control device, indicating improved color purity. The reduction in FWHM originates from the suppression of inhomogeneous broadening, which is primarily caused by energetic dispersion of trap states within the bandgap. In perovskite films, such traps capture charge carriers and facilitate radiative recombination at multiple trap levels, yielding broad emission profile.

Efficient trap passivation by K^+^ and EtO^-^ reduces these non-uniform recombination pathways, resulting in dominant band-edge emission and narrower spectral width. Figure S13b shows that PeKEtO achieves the highest luminance, significantly outperforming the control device, highlighting its enhanced brightness capabilities. Finally, Figure S13c demonstrates the highest EQE, highlighting its enhanced charge recombination efficiency and overall device performance.


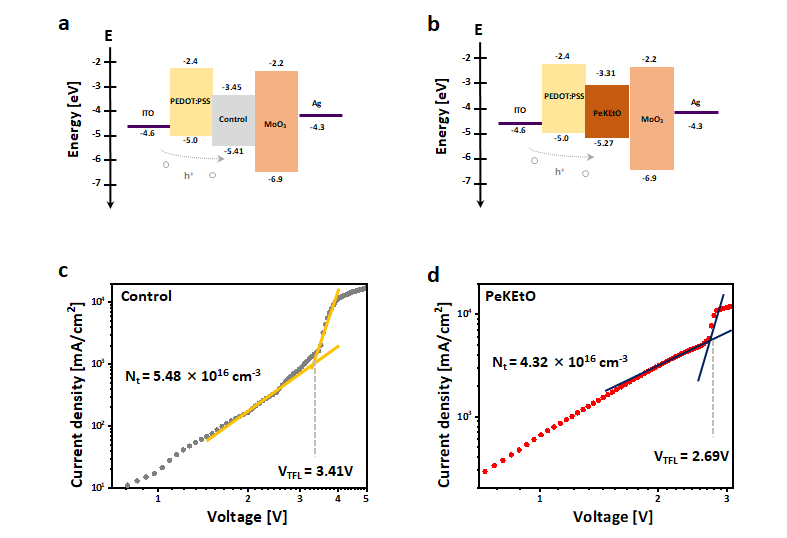


**Figure S14.** Band diagram of hole-only devices using (a) control and (b) PeKEtO films. Current density (*J*)-voltage (*V*) curves of (c) control- and (d) PeKEtO-based hole only devices.

Figures S14a and S14b illustrate the energy band alignment of hole-only devices (ITO/PEDOT:PSS/emissive layer/MoO_3_/Ag) for both the control and PeKEtO devices. Figures S14c and S14d display the current density-voltage (*J-V*) characteristics on a log-scale, with space-charge-limited current (SCLC) analysis.

The PeKEtO device exhibits a lower trap-filled limit voltage (V_TFL_) of 2.69V compared to the control device (3.41 V). From the fitted region, the extracted trap-density (N_t_) is reduced from 5.48 × 10^16^ cm^-3^ (control) to 4.32 × 10^16^ cm^-3^ (PeKEtO), confirming effective defect passivation through the OCOS strategy.


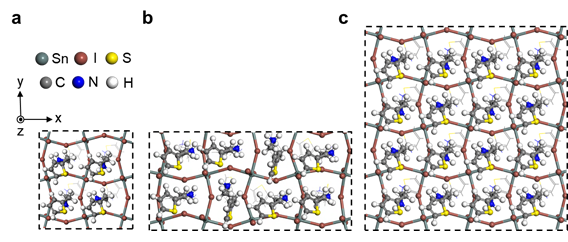


**Figure S15.** Top view images of the DFT-optimized structure of monolayer (TEA)_2_SnI_4_ models with different supercell sizes: (a) 2 × 2, (b) 2 × 4, and (c) 4 × 4 unit-cells.

**
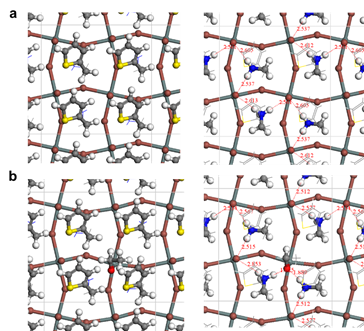
**

**Figure S16.** Optimized 2 × 2 unit-cell superstructures of (a) pristine (TEA)_2_SnI_4_ and (b) the corresponding model in which an I^-^ is replaced by EtO^-^.

The left figures show the arrangement of thiophene tail groups and the right ones show that of CH_2_-NH_3_^+^ head groups together with the hydrogen bond networks. The bottom TEA layer was removed for clarity. (Color scheme: Sn, charcoal; I, brown; C, gray; N, blue; S, yellow O, red; H, white). The hydrogen bonds between O and CH_2_-NH_3_^+^ moiety of TEA in PeEtO are shorter and stronger with bond lengths of 1.889 Å and 1.625 Å than those between I and CH_2_-NH_3_^+^ in pristine (TEA)_2_SnI_4_ with bond lengths of 2.605 Å and 2.561 Å.
